# A clinical study of traditional Chinese medicine prolonging the survival of advanced gastric cancer patients by regulating the immunosuppressive cell population: A study protocol for a multicenter, randomized controlled trail

**DOI:** 10.1097/MD.0000000000019757

**Published:** 2020-04-17

**Authors:** Xiaoting Pan, Heyun Tao, Mengjun Nie, Yuanjie Liu, Pan Huang, Shenlin Liu, Wei Sun, Jian Wu, Ting Ma, Anwei Dai, Jianwei Lu, Baorui Liu, Xi Zou, Qingmin Sun

**Affiliations:** a The Affiliated Hospital of Nanjing University of Chinese Medicine, Jiangsu Province Hospital of Chinese Medicine; b No. 1 Clinical Medical College, Nanjing University of Chinese Medicine, Nanjing, Jiangsu; c Traditional Chinese Medicine Hospital of Zhangjiagang; d Changzhou TCM Hospital; e Traditional Chinese Medicine Hospital of Kunshan; f Jiangsu Cancer Hospital; g Nanjing Drum Tower Hospital, China.

**Keywords:** a multicenter, gastric cancer, Jianpi Yangzheng Xiaozheng decoction, randomized controlled trail, traditional Chinese medicine

## Abstract

**Background:**

Gastric cancer (GC) is a common high-mortality disease, causing a serious social burden. Traditional Chinese medicine has been utilized to prevent and treat GC for many years but its effects remain unclear. The aim of our study is to elucidate the anti-tumor effects and the possible mechanism of Jianpi Yangzheng Xiaozheng decoction.

**Methods/design:**

This is a prospective, multicenter, randomized controlled trial continuing 1.5 years. Two hundred ten eligible patients will be randomly divided into 2 groups, the chemotherapy alone and the chemotherapy combined with JPYZXZ group at a ratio of 1:2. All patients will receive the treatment for 24 weeks and follow up for 1.5 years. The primary outcomes are one-year survival rate, progression-free survival, and overall survival (OS), while the secondary outcomes are immune related hematology test, objective response rate, tumor makers, traditional Chinese medicine syndrome points, fatigue scale, and quality of life scale. All of these outcomes will be analyzed at the end of the trail.

**Discussion:**

This study will provide the objective evidence for the efficacy and safety of Jianpi Yangzheng Xiaozheng decoction in advanced GC. Furthermore, it will be helpful to form a therapeutic regimen in advanced GC by the combination of traditional medicine and western medicine.

**Trail registration:** ChiCTR1900028147

## 1 Introduction

Gastric cancer (GC) is an important disease with high morbidity and mortality globally, which ranks the fifth most frequently diagnosed cancer and the third leading cause of cancer death. In 2018, over 1,000,000 new cases were diagnosed with GC and approximately 783,000 deaths (equating to 1 in 12 deaths worldwide).^[1]^ Most patients (>70%) with GC are diagnosed at the advanced stage,^[2]^ as a consequence, it shows a disappointing result.^[3,4]^ Accumulating evidence shows that only 5% of patients with metastatic cancer are still alive 5 years after diagnosis.^[5]^

In recent years, immune microenvironment has been increasingly valued for its crucial role in the process of GC onset and progression.^[6–8]^ Although surgical resection, chemotherapy, radiotherapy, biologic therapeutic agents, and other measures have been used to improve the therapeutic efficacy of GC,^[9]^ the prognosis is still dismal.^[10]^ Thus, it is important to explore new strategies to prolong the survival, improve the quality of life of the advanced GC patients. Fortunately, traditional Chinese medicine (TCM) has its unique advantages in preventing and treating GC^[11,12]^ and it also has an efficacy in improving immune function.^[13]^

The JPYZXZ decoction is a traditional Chinese herbal formulation created by Professor Shenlin Liu, the national famous Chinese Physician. JPYZXZ decoction is composed of 12 kinds of Chinese herbal medicine, as is shown in Table 1. Our previous studies^[14]^ have illustrated that JPYZXZ plays a critical role in improving cancer-related fatigue and prolonging survival in patients with advanced GC and it has been used to treat GC patients in Affiliated Hospital of Nanjing University of Chinese Medicine for many years. The aim of our present study is to explore the efficacy and safety of JPYZXZ decoction in advanced GC using a multicenter randomized controlled trail. Meanwhile, the changes of immunosuppressive cell population in peripheral blood of advanced GC patients will be detected to demonstrate the mechanism of JPYZXZ decoction.

**Table 1:**
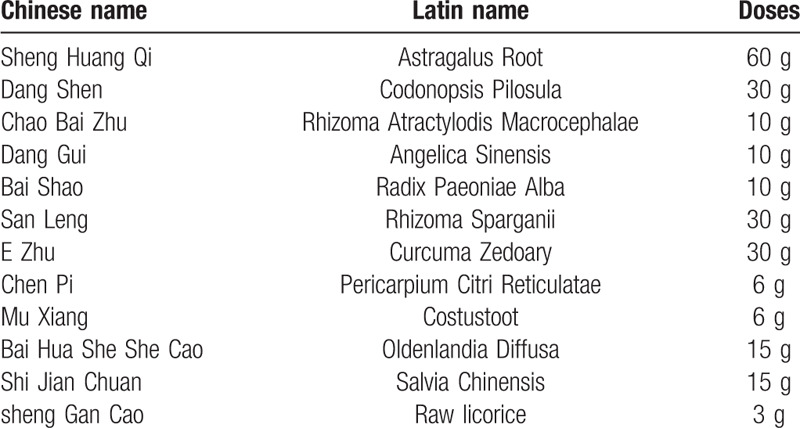
“Jianpi Yangzheng Xiaozheng decoction” (JPYZXZ) composition.

## 2 Methods/design

### 2.1 Design

**Figure 1:**
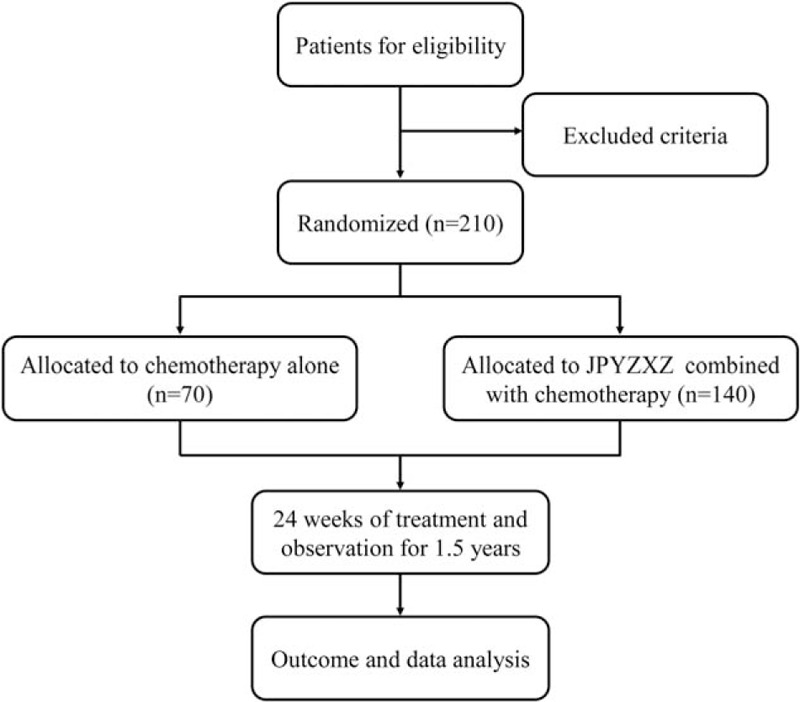
The study flowchart. The flowchart of enrollment, interventions and analysis.

This is a prospective, multicenter, randomized controlled trial lasting 1.5 years in 6 locations, including Affiliated Hospital of Nanjing University of Chinese Medicine, Jiangsu Cancer Hospital, Nanjing Drum Tower Hospital, Traditional Chinese Medicine Hospital of Kunshan, Changzhou TCM Hospital and Traditional Chinese Medicine Hospital of Zhangjiagang. A total of 210 eligibility patients with advanced GC will be enrolled in this trail. All the patients will be randomly assigned into 2 groups (in a ratio of 2:1): 140 in the treatment group (chemotherapy plus JPYZXZ granules) and 70 in the control group (chemotherapy alone). The study protocol has been approved by the Ethic Committee of Affiliated Hospital of Nanjing University of Chinese Medicine (Number 2019NL-166–02), and it will be conducted in accordance with the Declaration of Helsinki strictly. The study flowchart has been shown in Figure 1.

### 2.2 Randomization

After obtaining informed consent from eligible participants, researchers will upload the basic information. Then with the help of the third-party software Medroad Cloud, the random number will be given (https://sci.medroad.cn/). The participants will be randomly divided into the chemotherapy alone and chemotherapy plus JPYZXZ granules groups.

### 2.3 Recruitment and consent

A total of 210 patients with advanced GC will be recruited in this clinical trail. The purpose, benefits and potential risks of this trail and other information will be informed to the patients in detail. All the patients participating in this trail are entirely voluntary, and they can also withdraw at any time during the whole trail without any consequence. Written consent will be obtained from each eligibility patient before them participating in the study.

### 2.4 Inclusion criteria and exclusion criteria

#### 2.4.1 Inclusion criteria

(1) Patients with pathological diagnosis of GC;
(2) Patients with histologically or cytologically confirmed stage III after resection or IV, who cannot undergo radical surgery or postoperative recurrence and metastasis;
(3) Patients with criteria of TCM syndrome of Qi deficiency of spleen and stomach;
(4) KPS scores >60;
(5) Patients aged from 18 to 75-years-old;
(6) Patients with life expectancy of at least 6 months;
(7) Patients who provide written informed consent to participate in the study according to the GCP criteria;
(8) Childbearing age women whose urine pregnancy test is negative within 7 days before enrollment. All the patients and their spouse should abstinence all the time or at fixed periods during this trail or take at least 2 effective contraceptives. Abstinence or contraceptives still need to be used for 2 years after the trail.

#### 2.4.2 Exclusion criteria

(1) Patients who is unable to swallow oral medications including with digestive tract obstruction and jejunostomy;
(2) Patients with symptomatic brain metastasis or mental disorder;
(3) Patients with severe cardiovascular disease, chronic liver disease, kidney disease or blood disease;
(4) Patients whose laboratory examination before enrollment is abnormal according to the following criteria: Blood routine examination: ANC < 2.0 × 10^9^/L, Hb < 90 g/L, PLT < 80 × 10^9^/L, Renal function: Cr > 1.5 × upper normal limit (UNL); CCr < 50 ml/minute, liver function: TBil > 1.5 × UNL, ALT (SGPT) and AST (SGOT) > 1.5 × UNL;
(5) Patients who are pregnant, nursing;
(6) Patients who are substance abuse, or with clinical, mental, and social features which is interference for the study and informed consent;

### 2.5 Interventions

#### 2.5.1 The control group

Seventy patients in the control group will be treated with chemotherapy only, and all the chemotherapy regimens refer to the NCCN Guidelines (2019) of GC, specifics as follows (any one of them):

XELOX: Oxaliplatin 130 mg/m^2^, on day 1;
 Capecitabine 1000 mg/m^2^, on days 1 to 14, twice daily, every 3 weeks;
DS-1: Docetaxel 40 to 50 mg/m^2^, on day 1, every 2 weeks;
 S-1 40 to 60 mg/m^2^, on days 1 to 14, twice daily, every 3 weeks;
SOX: Oxaliplatin 85 mg/m^2^, on day 1, every 2 weeks;
 S-1 40 mg/m^2^, on days 1 to 14, twice daily, every 3 weeks;

#### 2.5.2 The treatment group

Total of 140 patients in the treatment group will be treated with chemotherapy combined with JPYZXZ decoction. The chemotherapy regimens will be the same as the control group and the JPYZXZ decoction, available in the forms of granules, should be taken at least 6 months, per bag twice daily, add boiled water in it and mix up it, 150 ml each time.

### 2.6 Outcome measures

The time-points of assessment is listed in Table 2.

**Table 2:**
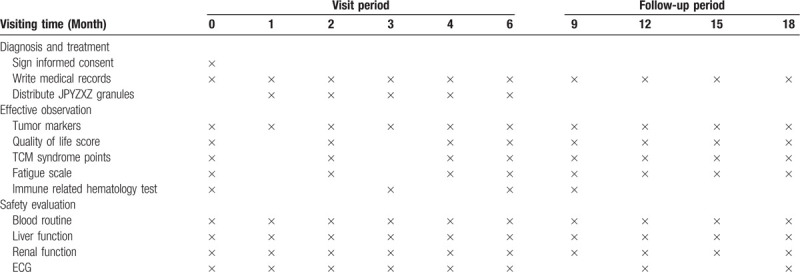
study schedule.

#### 2.6.1 Primary outcomes

The primary outcomes include 1-year survival rate, progression-free survival, and overall survival (OS). One-year survival rate = (the number of patients still alive after 1 year of follow-up / the total number of patients followed up) × 100%. Progression-free survival means the time from the start of treatment to recurrence or death due to any causes. OS is the time from the beginning of diagnosis to death for any causes. All of the indicators will be calculated at the end of the trail.

#### 2.6.2 Secondary outcomes

(1) Immune related hematology test: Flow cytometry will be used to analyze the differences between the immunosuppressive cell population in peripheral blood of the 2 groups before and after the treatment.
(2) Objective response rate: computed tomography will be used to monitor the size of tumor, and we will calculate the proportion of patients whose tumor shrink to a certain size and maintain for a period of time.
(3) Tumor makers: tumor markers like CEA, CA199 will be detected every 3 months to estimate whether the tumor recurrence or disease progression.
(4) TCM syndrome points: the evaluation criteria of TCM syndrome point is based on the “Chinese medicine clinical research of new drugs guiding principles”. Researchers will evaluate it every 2 months during the treatment, and then every 3 months until patients die. The efficacy (efficacy = (the integral value before treatment — the integral value after treatment) / the integral value before treatment × 100%) is assessed at 4 levels: clinical remission, symptoms and signs are significantly improved after treatment, and the efficacy ≥95%; significant improvement, symptoms, and signs are mostly improved after treatment and 70% ≤ efficacy < 95%; partial improvement, symptoms and signs are partially improved after treatment and 30% ≤ efficacy < 70%; no improvement, symptoms and signs are scarcely any improved after treatment or even worse, and the efficacy <30%. All of these results will be actual recorded in case report forms.
(5) Fatigue scale: Fatigue scale will be used to evaluate the changes of fatigue symptoms in patients with advanced GC before and after treatment. Researchers will estimate it every 2 months between treatment, and then every 3 months. All the information will be recorded in case report forms.
(6) Quality of life scale: EORTC QLQ-STO30, a questionnaire focused on symptoms, physical functions, and entire health situation, is used to evaluate the quality of life scale.

#### 2.6.3 Safety evaluation

Blood routine, Liver function, Renal function, and ECG will be monitored regularly to evaluate the safety of JPYZXZ decoction.

### 2.7 Adverse events

Although adverse effect of JPYZXZ has not been observed in preclinical studies, all the researchers should pay close attention to possible adverse reactions, such as vomiting. Any adverse events occurred during the trail should be recorded in “Adverse events form” and the treatment process and results also need to be recorded in detail. When serious adverse events appeared, researchers should terminate the study of the participant of this case and fill out the “serious adverse events form” in truth. Every adverse event needs to be reported to the ethic committee of Affiliated Hospital of Nanjing University of Chinese Medicine.

### 2.8 Sample size calculation

According to our previous study, the average survival time of advanced GC patients treated with chemotherapy is 257.92 ± 16.56 days. Considering that the minimum error clinical accepted is 3.5%, α is 0.05 and test efficiency is 80%. The sample size is calculated by the following formula: 
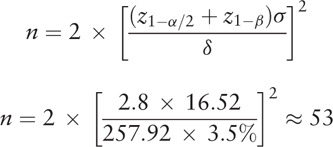


Considering 20% of the drop rate, allocating patients at a ratio of 2:1, the total sample size is 210 (140 in chemotherapy plus JPYZXZ group and 70 in chemotherapy alone group).

### 2.9 Statistical analysis

SPSS 18.0 and other statistics software will be used to analyze the outcomes. Quantitative data will be expressed as the mean ± standard deviation, and *T* test will be used to compare the data between 2 groups. Qualitative data will be calculated by chi-square test and Fisher test. Quality of life analysis will be performed by repeated measures of variance. The OS and median survival will be analyzed by Kaplan-Meier. All data will be analyzed with a 2-sided test and *P* ≤ .05 is considered statistically significant.

## 3 Discussion

In general, GC has the highest incidence in eastern Asia, especially in China, Korea, Mongolia, and Japan.^[15]^ Despite the improvement of the measures to treat GC, the OS and prognosis are still poor. Most patients occur recurrence and metastasis, suffering from various symptoms, such as pain, asthenia. Hence, effective measures for prevention and treatment of GC are attracting people's attention.

TCM is one of the most important complementary and alternative medicines, which has been clinically used for thousands of years. Numerous studies have proven that compared with chemotherapy alone, chemotherapy combined with TCM related to better OS and quality of life.^[16–18]^ JPYZXZ is an experience formula summarized by Professor Liu, Prof. Liu suggest that Pi-Xu (spleen deficiency) is the key factor in advanced GC and treatment should be based on disease and syndrome. On the one hand, JPYZXZ can invigorating spleen, strengthen the body resistance; on the other hand, it also can remove blood stasis; which conforms to the characteristic of advanced GC.

Therefore, this prospective, multicenter, randomized controlled trial will verify the efficacy and safety of TCM in treating advanced GC. At the same time, a therapeutic regimen, with the combination of traditional medicine and western medicine will be initially formed and exerting the synergistic effect of TCM in advanced GC.

### 3.1 Trail status

This clinical study started in July 2019 and is scheduled to end in June 2022. So far, we have received the ethical approval and registered at Chinese Clinical Trail Registry (www.chictr.org.cn ChiCTR1900028147). Patients recruitment is in progress when the manuscript submitted.

## Author contributions

**Conceptualization:** Xiaoting Pan

**Formal analysis:** Mengjun Nie, Yuanjie Liu

**Funding acquisition:** Xi Zou

**Investigation:** Jian Wu, Wei Sun, Pan Huang, Ting Ma, Anwei Dai, Jianwei Lu, Baorui Liu

**Project administration:** Xi Zou, Shenlin Liu

**Supervision:** Qingmin Sun

**Visualization:** Mengjun Nie

**Writing – original draft:** Xiaoting Pan

**Writing – review & editing:** Heyun Tao
